# Cold storage conditions modify microRNA expressions for platelet transfusion

**DOI:** 10.1371/journal.pone.0218797

**Published:** 2019-07-03

**Authors:** Nobuhiro Mukai, Yoshinobu Nakayama, Sachiyo Ishi, Takayuki Murakami, Satoru Ogawa, Kyoko Kageyama, Satoshi Murakami, Yuji Sasada, Jun Yoshioka, Yasufumi Nakajima

**Affiliations:** 1 Department of Anesthesiology and Critical Care, Kyoto Prefectural University of Medicine, Kyoto, Japan; 2 Department of Molecular, Cellular and Biomedical Sciences, CUNY School of Medicine, City College of New York, New York, NY, United States of America; 3 Department of Anesthesiology, Otokoyama Hospital, Kyoto, Japan; 4 Thermo Fisher Scientific, Life Technologies Japan Ltd., Life Solutions Group, Tokyo, Japan; 5 Department of Transfusion Medicine and Cell Therapy, Kyoto Prefectural University of Medicine, Kyoto, Japan; 6 Department of Anesthesiology and Critical Care, Kansai Medical University, Osaka, Japan and Outcomes Research Consortium, Cleveland, OH, United States of America; Mayo Clinic Minnesota, UNITED STATES

## Abstract

MicroRNAs (miRNAs) are small RNA molecules that modulate gene and protein expression in hematopoiesis. Platelets are known to contain a fully functional miRNA machinery. While platelets used for transfusion are normally stored at room temperature, recent evidence suggests more favorable effects under a cold-storage condition, including higher adhesion and aggregation properties. Thus, we sought to determine whether functional differences in platelets are associated with the differential profiling of platelet miRNA expressions. To obtain the miRNA expression profile, next-generation sequencing was performed on human platelets obtained from 10 healthy subjects. The miRNAs were quantified after being stored in three different conditions: 1) baseline (before storage), 2) stored at 22°C with agitation for 72 h, and 3) stored at 4°C for 72 h. Following the identification of miRNAs by sequencing, the results were validated at the level of mature miRNAs from 18 healthy subjects, by using quantitative polymerase chain reaction (qPCR). Differential expression was observed for 125 miRNAs that were stored at 4°C and 9 miRNAs stored at 22°C as compared to the baseline. The validation study by qPCR confirmed that storage at 4°C increased the expression levels (fold change 95% CI) of mir-20a-5p (1.87, p<0.0001), mir-10a-3p (1.88, p<0.0001), mir-16-2-3p (1.54, p<0.01), and mir-223-5p (1.38, p<0.05), compared with those of the samples stored at 22°C. These results show that miRNAs correlate with platelet quality under specific storage conditions. The data indicate that miRNAs could be potentially used as biomarkers of platelet quality.

## Introduction

Platelet transfusion is an essential medical treatment for patients with quantitative and qualitative disorders of platelets [1]. Normally, it is recommended that platelets be stored at room temperature (20‒24°C) with gentle agitation for up to 5 days. However, these conditions increase the risk of transfusion-related infection due to bacterial contamination and growth [2, 3]. Moreover, it has been reported that room temperature storage induces impairment of hemostatic function [4, 5].

By far, cold (4°C) platelet storage has not been established as a standard method, mainly because cold temperature shortens the lifespan of platelets in the circulation [6, 7]. However, cold-storage conditions have been reported to promote favorable effects on hemostatic function [8, 9]. Under such conditions, platelets are activated with greater P-selectin secretion and Glycoprotein Ib surface expression along with an increase in thromboxane A2 production [10–12]. These functional enhancements in platelets by cold temperature lead to reduced bleeding time [8, 13], thereby being advantageous for patients with hemorrhage.

MicroRNAs (miRNAs) are small functional RNA molecules composed of 18 to 25 nucleotides. The miRNAs negatively control gene expression by transcriptional or post-transcriptional repression. Platelets contain abundant RNAs including miRNAs and have fully functional mRNA splicing machinery [14–16]. Platelet miRNAs are capable of regulating platelet mRNA and protein expression levels [17]. Interestingly, it has been reported that the miRNA profile of platelets is related to cellular damage under room-temperature storage conditions [18].

Based on these insights, we sought to characterize the differential profiling of platelet miRNA expression between room temperature and cold temperature storage conditions. To accomplish this, we performed comprehensive analyses of miRNAs in normal human platelets using next-generation sequencing.

## Materials and methods

### Ethics, subjects, and consent

This prospective observational study was approved by the Institutional Review Board of Kyoto Prefectural University of Medicine. Written informed consent was obtained from the healthy volunteers. Participants fulfilling any of the following criteria were excluded from the study: receiving a drug therapy, hematocrit <35%, body weight <40 kg, and having donated blood within three months. None of the donors were smokers, alcoholic, or drug users.

### Platelet preparation and storage

Whole blood was obtained by venipuncture and pooled in the Sepacell Integra system (Kawasumi Laboratories, Inc., Tokyo, Japan). The packed whole blood was centrifuged at 150 *g* for 12 min at 22°C. The supernatant containing platelet-rich plasma was gently isolated on a separation stand. The isolated platelet-rich plasma was stored in individual storage bags.

All procedures were performed aseptically. Platelets were prepared and stored at two different conditions: 1) at 22°C with agitation for 72 h (room temperature) or 2) at 4°C without agitation for 72 h (cold temperature). Then, the platelets were rewarmed to 37°C for 10 min prior to use. Washed platelet suspensions were prepared as previously described [19]. Results were compared among three groups: baseline (Day 0), room temperature after 72 h (Day 3 room), and cold temperature after 72 h (Day 3 cold).

### Isolation and sequencing of miRNA

Platelet miRNAs were extracted using the RNeasy Plus Mini Kit and RNeasy MinElute Cleanup Kit (Qiagen, Venlo, Netherlands) according to the manufacturer’s instructions. The miRNA size range was checked and quantified using Bioanalyzer and the Agilent Small RNA kit (Agilent Technologies, CA). We used 2 ng of RNA for each sample. Complementary DNA (cDNA) libraries were constructed with the Ion Total RNA-seq kit v2 (Thermo Fisher Scientific, MA) for RNA libraries. The purities and concentrations were measured by Bioanalyzer with the Agilent DNA kit. Sequencing of the miRNA library was performed on a 318 chip using an Ion PGM system (Thermo Fisher Scientific, MA). All raw reads were automatically trimmed to remove adaptors and aligned to miRBase v21 (hg19 CRCh37) using Torrent Suite v5.0.3 (Thermo Fisher Scientific) with default parameters. Small and non-coding RNAs were classified according to gene types.

### Quantitative polymerase chain reaction (qPCR)

We performed qPCR to determine the expression levels of mature sequences of miRNA. The miRNAs were extracted as described above. Complementary DNA was produced using the ABI Taqman advanced microRNA complementary DNA synthesis kit (Thermo Fisher Scientific, MA). A small RNA molecule, 5´-UUUGGAUUGAAGGGAGCUCUA-3´, was spiked into samples as a spike-in control for miRNA analyses. A qPCR analysis was conducted by using Step One Plus (Thermo Fisher Scientific, MA). Forty-cycle amplification (Cycle threshold [Ct] values = 40) was employed for the cycle threshold. The 2^–ΔΔCt^ method was used to analyze expression values.

### Statistical analysis

We estimated that ten patients per group were an appropriate sample size for miRNA sequencing, based on previous reports [17, 20]. Analyses of digital gene expression by CLC Genomic Workbench version 8.5.1 (Qiagen, Venlo, Netherlands) were performed to compare the results of miRNA sequencing. False discovery rate-corrected *P*-values (FDR-*P*) below 0.05 were considered significant.

Sample size estimation for the qPCR validation was based on α = 0.05, β = 0.2, and effect size = 0.5 with three groups using G*power 3.1.9.2 (http://www.gpower.hhu.de/). At least 14 participants were required. Accordingly, we enrolled 18 participants in the study. qPCR results were compared by one-way analysis of variance (ANOVA) followed by Tukey’s post-hoc test and are expressed as mean with standard deviations. All statistical analyses were performed with Stat-Flex version 6.0 (Artech Co., Ltd., Osaka, Japan) and Graph Pad Prism 7.00 (Graphpad Software). Results with *P*-values less than 0.05 were considered significant.

## Results

### miRNA sequencing

We isolated miRNAs from 10 participants. The basic characteristics of these individuals were: six males and four females, age 34 ± 4.8 years, height 167 ± 8.8 cm, weight 64 ± 12 kg, and hematocrit 41 ± 2.5%.

One hundred and twenty-five miRNAs were identified as differentially expressed between baseline (Day 0) and cold storage (Day 3 cold). Nine miRNAs were identified as differentially expressed between baseline (Day 0) and room temperature storage (Day 3 room), and 68 miRNAs were identified between room temperature (Day 3 room) and cold (Day 3 cold) storage conditions (S1 and S2 Figs). The top 20 miRNAs with the most significant differences are shown in Table 1. The miRNAs are arranged in ascending order of the FDR-*P*-value (Day 0 vs. Day 3 cold).

**Table 1:** Differentially expressed miRNAs under three different conditions.

microRNA ID	Mean read counts			Day 0 vs. Day 3 cold		Day 0 vs. Day 3 room		Day 3 room vs. Day 3 cold	
	Day 0	Day 3 room	Day 3 cold	log _2_ fold change	FDR–*P* value	log _2_ fold change	FDR–*P* value	log _2_ fold change	FDR–*P* value
mir-339	2799	1406	914	-1.92	< E-40	0.55	0.002	-1.15	5.62E-09
mir-1307	3983	2096	1469	-1.68	< E-40	0.53	0.048	-1.05	3.40E-07
mir-20a	25311	30901	73074	1.26	3.82E-38	-0.76	7.94E-07	0.69	1.98E-11
mir-16-2	11264	13261	31563	1.24	5.77E-30	-0.63	3.89E-05	0.69	1.95E-09
mir-16-1	11939	13889	33378	1.24	2.09E-29	0.56	0.0001	0.71	1.35E-09
let-7f-2	10579	10583	29367	1.23	1.35E-25	0.31	0.22	0.92	2.42E-13
let-7f-1	9673	9651	26766	1.22	6.05E-25	0.31	0.22	0.91	2.77E-13
mir-18a	9995	9929	21775	0.86	2.25E-22	0.28	0.06	0.57	9.27E-10
mir-23a	45	24	16	-1.76	3.01E-20	-0.59	0.07	-1.17	4.96E-08
let-7g	15077	13976	32563	0.86	1.65E-19	0.21	0.46	0.65	7.32E-11
mir-374b	4089	3949	9104	0.92	3.99E-19	0.29	0.16	0.63	6.73E-09
mir-15a	10374	10342	26408	1.09	3.33E-17	0.33	0.23	0.76	2.67E-08
mir-139	1687	1950	5001	1.36	2.06E-16	0.45	0.18	0.91	1.59E-07
mir-126	40204	39748	95026	0.97	2.58E-16	0.30	0.24	0.67	8.21E-08
let-7a-1	6934	5954	14651	0.81	9.46E-16	0.10	> 0.99	0.72	1.52E-11
let-7a-2	6960	5980	14670	0.81	9.50E-16	0.10	> 0.99	0.71	1.52E-11
let-7a-3	6853	5893	14481	0.81	1.35E-15	0.10	> 0.99	0.72	1.62E-11
mir-181c	579	357	300	-1.17	1.54E-14	-0.30	0.58	-0.86	7.31E-08
mir-30b	13610	12185	29500	0.86	4.67E-13	0.11	> 0.99	0.74	1.43E-09
mir-671	100	73	36	-1.75	9.21E-13	-0.21	> 0.99	-1.54	1.74E-09

The table shows the top 20 miRNAs that are differentially expressed under three different conditions (Day 0; baseline, Day 3 room; 22°C storage with agitation for 72 h, Day 3 cold; 4°C storage without agitation for 72 h). Mean read counts are expressed as non-normalized read counts.

**Fig 1:**
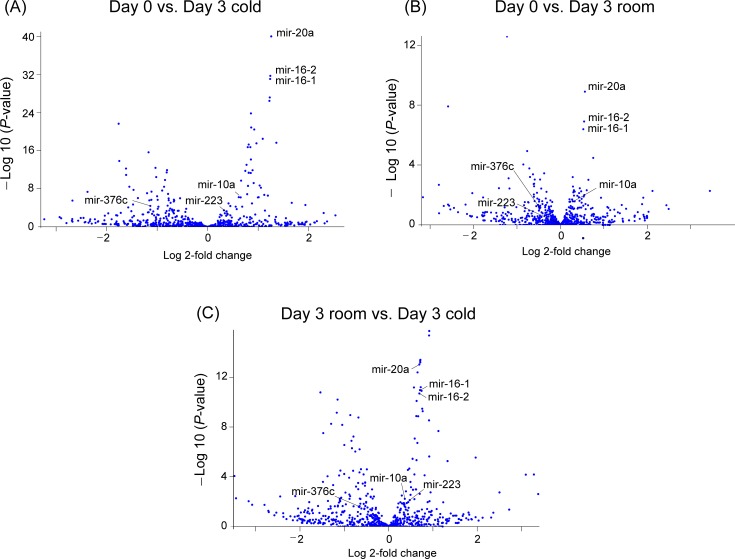
Volcano plots comparing changes in the expression of miRNAs between platelets exposed to baseline (Day 0), room-temperature (Day 3 room), and cold-storage (Day 3 cold) conditions.

A volcano plot and heat map of the comparisons are shown in Fig 1 and S3 Fig, respectively, revealing prominent changes in mir-20a, mir-16-2, and mir-16-1.

The changes in levels of mir-20a, mir-16-2, and mir-16-1 were prominent in the comparison. mir-10a, mir-223, and mir-376c, which were focused on in the validation study by qPCR, are included in these plots. The miRNAs were recognized as stem-loop sequences. miRBase 21 was used as database (http://www/mirbase.org/).

### Validation study by qPCR

Next, we sought to focus on several candidate miRNAs as critical regulators of platelet functions and to verify the changes in their expression levels as mature sequences of miRNA using qPCR. We isolated miRNAs from 18 participants, among whom 12 were males and six were females, age 32 ± 5.5 years, height 168 ± 7.2 cm, weight 62 ± 10 kg, and hematocrit 41 ± 2.1%. Five samples overlapped with those used for miRNA sequencing. We measured miRNA levels of the three most highly expressed miRNAs (mir-20a, mir-16-1, and mir-16-2) that were induced by cold temperature. In addition, the levels of miRNAs that had been previously shown to regulate platelet membrane proteins were measured. These include mir-10a, which regulates the expression of glycoprotein 1b (GP1b) [21], and mir-223, which controls the expression of putative G protein coupled receptor (P2Y12) [22, 23]. We also measured the level of mir-376c, which modifies the protease activated-receptor 4 (PAR4) signaling via phosphatidylcholine transfer protein (PCTP) [24].

**Fig 2:**
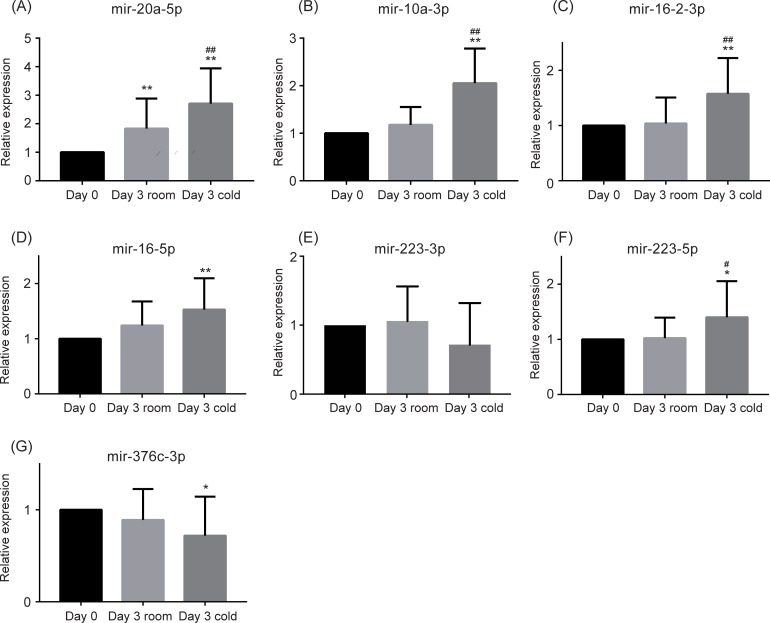
Differential microRNA profiling with qPCR. Data represent the mean + standard deviation (SD). * *P* <0.05, ** *P* <0.01 vs. Day 0. ^#^ *P* <0.05, ^##^ *P* <0.01 vs. Day 3 room.

qPCR results are shown in Fig 2 and Table 2. Several samples had undetermined Ct values (Ct values >40). Thus, mir-20a-3p, mir-10a-5p, mir-16-1-3p, and mir-376c-5p were excluded from the three-group comparison for statistical accuracy. The expression levels of mir-20a-5p (Fig 2A), mir-10a-3p (Fig 2B), mir-16-2-3p (Fig 2C), mir-16-1-5p/mir-16-2-5p also known as mir-16-5p (Fig 2D), mir-223-5p (Fig 2F), and mir-376c-3p (Fig 2G) were found to be changed in the cold storage conditions (Day 3 cold) compared to baseline (Day 0). Among these, levels of mir-20a-5p (Fig 2A), mir-10a-3p (Fig 2B), mir-16-2-3p (Fig 2C), and mir-223-5p (Fig 2F) were significantly increased under cold temperature (Day 3 cold) compared to that at room temperature (Day 3 room). The differential expression levels of other miRNAs measured did not reach statistical significance between the cold (Day 3 cold) and room temperature (Day 3 room) samples.

**Table 2:** Differentially expressed microRNAs in three-group comparison using qPCR.

microRNA ID	Day 0 vs. Day 3 cold		Day 0 vs. Day 3 room		Day 3 room vs. Day 3 cold	
	Fold change	*P*-value	Fold change	*P*-value	Fold change	*P*-value
	(95% CI)		(95% CI)		(95% CI)	
mir-20a-5p	2.71 (1.96–3.45)	< 0.0001	1.83 (1.20–2.47)	0.0009	1.87 (1.58–2.17)	< 0.0001
mir-10a-3p	2.05 (1.67–2.43)	< 0.0001	1.18 (0.80–1.56)	0.5	1.88 (1.50–2.26)	< 0.0001
mir-16-2-3p	1.58 (1.18–1.97)	0.0041	1.04 (0.75–1.32)	0.94	1.54 (1.22–1.85)	0.0011
mir-16-5p	1.53 (1.19–1.87)	0.0027	1.24 (0.98–1.50)	0.077	1.29 (0.87–1.71)	0.2
mir-223-3p	–	0.95	–	0.95	–	0.13
mir-223-5p	1.40 (1.00–1.80)	0.046	1.24 (0.80–1.25)	0.95	1.38 (1.04–1.72)	0.03
mir-376c-3p	0.72 (0.46–0.98)	0.03	0.89 (0.69–1.09)	0.37	0.83 (0.55–1.10)	0.28

Day 3 room and Day 3 cold represent the following storage conditions: Day 3 room; 22°C with agitation for 72 h and rewarmed to 37°C for 10 min, Day 3 cold; 4°C with standing and rewarmed to 37°C for 10 min.

## Discussion

In this study, we performed next-generation sequencing of miRNA in normal human platelets to identify miRNAs that might be associated to platelet functions depending on the storage condition of the platelets. Then, qPCR was conducted to confirm mature miRNA profiling for markedly changed miRNAs. We found mir-20a-5p, mir-10a-3p, mir-16-2-3p, and mir-223-5p as potentially important key regulators that are sensitive to storage condition of human platelets used for transfusion.

Platelets are normally denucleated because they originate from the cytoplasmic fragmentation of megakaryocytes, suggesting a low capability of synthesizing new miRNAs. The lifespan of platelet miRNAs is relatively short owing to the rapid turnover [25, 26]. Thus, most platelet miRNA expression levels are expected to decrease over time when platelets are stored under room temperature [18, 27, 28]. However, specific miRNA expression levels, such as those of let-7b/mir-16 [27], mir-326 [29], mir-127/mir320a [18], and mir-570 [30], can increase under the same conditions. Pontes et al. reported that approximately 22% of miRNAs undergo decrease in their expression levels from day 1 to day 5, whereas other miRNAs can increase even after seven days following blood collection [18]. Interestingly, it has been reported that some miRNAs are also upregulated in red blood cells (anucleate cells) during 4°C storage [31]. It remains unclear whether the observed differential miRNA expression is universally due to degradation of miRNAs as a result of storage in all types of stored tissue specimens. This is particularly important because anucleate cells such as platelets and red blood cells should not actively synthesize miRNAs, indicating that degradation may be a major contributor to increases in miRNAs. Another report has demonstrated that miRNA degradation can be observed to some extent in formalin-fixed paraffin-embedded tissues [32]. Accordingly, it is possible that differential miRNA expression can be seen in other cell types under storage at 4°C and that this is not necessarily specific to platelets.

Some potential mechanisms are suggested to explain how miRNA expression might be increased in human platelets. The miRNA precursors associated with aging are converted to mature miRNAs by RNA-editing enzymes [33]. The RNA precursors can be cleaved into small RNA fragments with a regulatory capacity to suppress protein translation in response to stress [34]. Another possibility is that cold-inducible RNA-binding protein, such as RBM3, regulating miRNA biogenesis at the Dicer step, is markedly stimulated during the process of being cooled at 4°C and contributes to increased miRNA expressions [35]. These mechanisms seem reasonable because platelets have a rapid turn over of miRNAs [36] and expression levels of platelet miRNAs increase in a short time under marked stress [17].

Importantly, platelet miRNAs are known to regulate platelet protein expressions [21, 22, 24]. For instance, a sequential increase in mir-326 expression during the storage period down-regulates the expression of the anti-apoptotic gene *BCL-XL* and results in platelet apoptosis [29]. The increased mature RNA expression levels of mir-223 and mir-376c, which are observed in this study as well, decrease the protein levels of P2Y_12_ and PAR4 receptors (predicted by TargetScanHuman 7.2; http://www.targetscan.org/vert_72/). mir-20a negatively regulates the expression of NLRP3-inflammasomes by targeting thioredoxin-interacting protein (*TXNIP*) mRNA [37]. The elevation in mir-20a suppresses GPlb and glycoprotein Vl (GPVl) expressions through an increase in thioredoxin activity by inhibiting the function of TXNIP [38].

There are several limitations to this study. First, we only focused on some of the well-known miRNAs, such as mir-20a-5p, to verify the changes in expression levels of mature sequences using qPCR. Thus, we could have missed the opportunity to identify other potentially important miRNAs. Additionally, it may be useful to analyze several miRNAs that are not expected to be differentially expressed as a control group. Second, the methods that we used to identify miRNAs during room-temperature storage were different from those in previous studies [18, 27, 28]. The technical differences include: 1) collected platelets were stored as platelet-rich plasma, 2) the concentration of platelets was lower than that of typical platelet usage, 3) platelets were stored in citrate-phosphate-dextrose solution with adenine (CPDA), and 4) leukocyte counts (0.28 ± 0.14 × 10^3^ /μL, normal range: 4.0‒12.0 × 10^3^ /μL) in our samples were slightly concentrated than those in typical platelet usage due to a leukocyte-removing filter to prevent platelet adsorption during sample preparation. Other factors include cellular conditions affected by nutritional status of blood donors and exposure to environmental factors [39, 40]. Racial differences have also been reported to influence the platelet miRNA-mRNA-protein axis [24]. Third, miRNA sequence reads were aligned to miRBase v21 in the present study. We used miRBase v21 to prevent misalignment to regions of the genome other than those that code for miRNAs. Thus, the results may have differed had the entire human genome been used as an alignment reference. Fourth, we did not confirm that the RNA-seq estimations are in agreement with qPCR findings for both stable endogenous control and experimental samples. This is because obtaining optimal endogenous controls for normalization is challenging in the case of circulating miRNAs such as those in platelets and erythrocytes. Therefore, we used exogenous spike-in controls for normalization, which have been validated previously [41, 42]. Fifth, mir-20a-3p, mir-10a-5p, mir-16-1-3p, and mir-376c-5p were excluded from the three-group comparison, because several samples had undetermined Ct values (Ct values >40) [20]. Our results could have been different if we had modified the Ct value threshold for detection of these miRNAs. Finally, this study had a limited sample size. Ideally, these results should be validated in a larger cohort.

Even though cold-storage conditions increase platelet hemostatic function [8, 9], prolonged storage may cause potential problems in platelet transfusion. The capacity for translation into protein may be lower under cold storage conditions. However, after rewarming, the miRNA-mRNA-protein axis recovers [43, 44]. This recovery may be associated with decreased membrane protein expression [17] and other unknown biological effects. As a result, cold-storage platelets are preferred for transfusion into patients with major bleeding or minor bleeding that proceeds to major bleeding in the case of trauma or major surgeries. Thus, the greater procoagulant properties accompanied by differential miRNA expressions may be specifically beneficial during trauma resuscitation. This is because the platelets are consumed for hemostasis immediately after transfusion. However, clinical use of cold-stored platelets should be carefully considered until the precise molecular biological effects are fully characterized.

In conclusion, we identified 125 miRNAs, by miRNA sequencing, as differentially expressed under cold storage conditions. Among them, mir-20a-5p, mir-10a-3p, mir-16-2-3p, and mir-223-5p were validated as mature miRNAs, using qPCR. These results provide insights into the molecular mechanism by which storage conditions result in functional differences in human platelets. Further research is warranted to improve our knowledge on the association between differentially-expressed miRNAs and the quality of platelets used for transfusion in daily medical practice.

## Supporting information

S1 Fig All 125 miRNAs in platelets are differentially expressed under three different storage conditions. The miRNAs are arranged in ascending order of the FDR-*P*-value (Day 0 vs.Day 3 cold). Day 0 represents baseline control. Day 3 room represents storage at 22°C with agitation for 72 h and rewarming to 37°C in 10 min. Day 3 cold represents storage at 4°C without agitation for 72 h and rewarmed to 37°C in 10 minutes. Mean read counts are expressed as non-normalized read counts.
(EPS)

S2 Fig The top 20 most abundant miRNAs that are differentially expressed under three different conditions. The miRNAs are arranged in descending order of Day 3 cold mean read counts. Day 0 represents baseline control. Day 3 room represents storage at 22°C with agitation for 72 h and rewarming to 37°C in 10 min. Day 3 cold represents storage at 4°C without agitation for 72 h and rewarmed to 37°C in 10 minutes. Mean read counts are expressed as non-normalized read counts.
(EPS)

S3 Fig Heat map comparisons of miRNAs in platelets between three different storage conditions. The right panel shows the entire heat map. An area of high expression is expanded in the left panels. Day 0 represents baseline control. Day 3 room represents the storage condition of 22°C with agitation for 72 h and rewarming to 37°C in 10 min. Day 3 cold represents the storage condition of 4°C without agitation for 72 h and rewarmed to 37°C in 10 min. Redder areas show higher expression levels of miRNAs, whereas bluer areas show lower expression levels of miRNAs. Note that mir-16-1 and mir-16-2 show higher expression levels at Day 3 cold, and lower expression levels at Day 0.
(EPS)
